# The Diagnostic Utility of the Triptorelin Stimulation Test Compared to the Standard Gonadotropin-Releasing Hormone Stimulation Test in Children with Idiopathic Central Precocious Puberty

**DOI:** 10.3390/diseases13110370

**Published:** 2025-11-12

**Authors:** Giorgio Sodero

**Affiliations:** 1 Pediatric Unit, Perrino Hospital, 72100 Brindisi, Italy; giorgio.sodero@hotmail.it; 2 Pediatric Endocrinology Unit, Perrino Hospital, 72100 Brindisi, Italy

**Keywords:** central precocious puberty, GnRH stimulation test, triptorelin, luteinizing hormone, pediatric endocrinology, pubertal disorders

## Abstract

Background: Central precocious puberty (CPP) is diagnosed through a combination of clinical, auxological, and biochemical parameters, with pharmacological stimulation tests considered the diagnostic gold standard. In recent years, triptorelin, a long-acting Gonadotropin-Releasing Hormone (GnRH) analog, has been increasingly adopted in clinical practice due to limited availability of native GnRH. Objective: To compare the clinical, auxological, and hormonal profiles of girls diagnosed with idiopathic CPP using either the classical GnRH stimulation test or the triptorelin test. Methods: This retrospective study included 136 female patients diagnosed with CPP and followed for at least two years at a single pediatric endocrinology unit. Of these, 101 underwent a GnRH stimulation test, and 35 were assessed using the triptorelin test. Baseline and stimulated hormonal parameters, growth data, and IGF-1 levels were collected. A multivariate linear regression model was used to explore the influence of age, test type, and other covariates on the LH peak response. Results: Anthropometric and baseline hormonal parameters were comparable between the two groups. The LH peak was significantly higher in the GnRH group (9.8 ± 3.1 IU/L at 60 min) than in the triptorelin group (6.8 ± 2.4 IU/L at 4 h). FSH levels were also significantly lower following triptorelin stimulation (*p* = 0.004), while the LH/FSH ratio did not differ significantly. Multivariate analysis confirmed that triptorelin was associated with a lower LH peak (β = −2.2, *p* = 0.008), particularly in younger patients, with a significant interaction between age and test type (β = 0.6, *p* = 0.022). Conclusions: Both GnRH and triptorelin stimulation tests are valid tools for CPP diagnosis. However, the GnRH test appears to elicit a more robust LH response, especially in younger patients, whereas the triptorelin test is associated with delayed and lower LH peaks.

## 1. Introduction

Central precocious puberty (CPP) is a clinical condition characterized by the early onset of secondary sexual characteristics [1,2], occurring before the age of 8 years in girls and before 9 years in boys [3,4]. The epidemiology of CPP shows notable differences based on geography, ethnicity, and socioeconomic factors [1]. Overall, CPP is relatively rare, with an estimated prevalence ranging from 1 in 5000 to 1 in 10,000 children [1,2,3,4]. The condition is significantly more common in females than in males, with a reported female-to-male ratio of approximately 10:1 [1,2]. Most cases of CPP are idiopathic, especially in girls, although identifiable causes such as central nervous system abnormalities, genetic mutations, and exposure to environmental endocrine disruptors have been documented [3,4].

Although the diagnosis of CPP is standardized according to various international guidelines [5,6], it still requires a multidisciplinary evaluation of patients [7]. This is because, while the pharmacologically stimulated increase in gonadotropins is widely considered the diagnostic gold standard, there is no universally accepted criterion for interpreting the different stimulation tests [1,2,3,4,5,6,7,8]. In Italy, for example, the LH cutoff value during stimulation tests ranges between 3.3 and 5 IU/L [7]. However, several studies in the literature have shown that intermediate cutoff values may already be sufficient to establish a diagnosis of CPP [8,9].

In recent years, the diagnostic process has become more complex due to the temporary unavailability of GnRH for stimulation testing [10]. This shortage has necessitated the use of alternative stimulation tests, such as the one employing triptorelin, a molecule already widely used in the treatment of CPP [11].

Although the triptorelin stimulation test has become widely used in recent years [12], it is not considered the gold standard for the diagnosis of central precocious puberty; nonetheless, it currently represents the most frequently employed stimulation test for CPP diagnosis in our country [7,8].

The aim of this retrospective study is to analyze the clinical, auxological, and hormonal characteristics of patients with idiopathic central precocious puberty, by comparing a cohort diagnosed using the classical GnRH stimulation test with another cohort evaluated through the triptorelin test.

## 2. Materials and Methods

We analyzed the clinical, auxological, and hormonal parameters of pediatric patients diagnosed with central precocious puberty, who were under periodic follow-up at the Pediatric Endocrinology Unit of our hospital. All patients underwent the standard diagnostic protocols for CPP, including auxological evaluation and baseline and stimulated hormonal assays. In accordance with our current guidelines, all patients exhibited a peak LH level greater than 5 IU/mL, as assessed by either the GnRH stimulation test or the triptorelin test.

Subsequently, all patients were treated with triptorelin [1] to halt the progression of secondary sexual characteristics and were followed regularly for a minimum period of at least one year. The administration of triptorelin is authorized for the treatment of CPP on a monthly or every three months basis [1,2,3,4,5,6,7,8,9,10]; the decision regarding the frequency of treatment, as well as the dosage, is at the discretion of the clinician responsible for the patient. This decision takes into account the patient’s characteristics, the rate of progression of secondary sexual characteristics, and, importantly, the preferences of the patient and her family. In both the monthly and three-month administrations, the same drug is used, with only the dosage differing.

Below, we present the main characteristics of the stimulation tests employed.

### 2.1. GnRH Test

Despite variations in diagnostic approaches, all major guidelines recognize the GnRH stimulation test as a pivotal tool for confirming CPP [5,6]. This test involves the intravenous administration of up to 100 µg of GnRH, followed by serial measurements of luteinizing hormone (LH) and follicle-stimulating hormone (FSH) at 0, 30, 60, 90, and 120 min post-injection [8].

A diagnosis of CPP is supported by a peak LH value exceeding either 3.3 IU/L or 5.0 IU/L [4,5,6,7,8,9], depending on the specific guideline referenced. Additionally, the LH/FSH ratio after stimulation, typically considered significant when greater than 0.6 or 1, provides further diagnostic support [1,2]. Once the diagnosis of CPP is established, patients typically initiate treatment with GnRH analogs [10,11,12,13]. These are administered intramuscularly at a standard dose of 3.75 mg every 3–4 weeks and continued until the child reaches the appropriate age for physiological pubertal development [7,8,9].

### 2.2. Triptorelin Test

In recent years, the triptorelin stimulation test has emerged as a practical and widely adopted alternative to the classical GnRH test for the diagnosis of CPP [11], particularly in settings where native GnRH is not readily available. Triptorelin, a long-acting GnRH analog, exerts a similar stimulatory effect on the hypothalamic-pituitary-gonadal axis but with a delayed peak response due to its prolonged half-life [11,12]. The test typically involves the administration of 100 µg of triptorelin, followed by measurement of LH and FSH at baseline and 4 h post-injection. In some protocols, additional sampling at 2, 3, or 24 h may be performed to capture the individual’s peak response.

A pubertal LH peak above 5 IU/L at 4 h, along with an LH/FSH ratio greater than 0.6 or 1, is generally considered indicative of CPP. However, unlike the classical GnRH test, standardized cutoff values for the triptorelin test remain less well-defined; for example, the test involving subcutaneous administration of triptorelin has a cutoff value of 15 IU/mL, which differs significantly from the cutoff used for intramuscular administration. In our clinical practice, the triptorelin test has proven to be a reliable and well-tolerated tool for the diagnostic workup of suspected CPP, particularly when logistical constraints limit access to the standard GnRH test. Nonetheless, the delayed timing of hormonal peaks and the comparatively lower magnitude of LH response must be taken into account when interpreting the results. The use of the Triptorelin test is, however, a recent development; therefore, while there is strong evidence and well-established guidelines regarding the GnRH test, it is possible that the protocol for administering this test may undergo modifications in the coming years. Furthermore, according to current protocols, the test is lengthy and can be challenging to tolerate for both the patients and their families.

### 2.3. Additional Information

Statistical analyses were conducted using IBM SPSS Statistics, version 25.0 (IBM Corp., Armonk, NY, USA). Continuous variables were summarized as mean ± standard deviation (SD) to provide an indication of central tendency and variability within the data. Categorical variables were presented as frequencies and corresponding percentages to describe the distribution of categories within the sample. The normality of continuous data distribution was assessed using the Shapiro–Wilk test, which determines whether the data deviates significantly from a normal distribution. For comparisons between groups, parametric tests were applied when data met normality assumptions; specifically, the independent samples *t*-test was used to compare means of normally distributed continuous variables. In cases where the normality assumption was violated, non-parametric alternatives such as the Mann–Whitney U test were employed to compare median values between groups. For categorical variables, group differences were evaluated using the chi-square test to assess associations between categorical variables. When expected cell counts were small or assumptions for the chi-square test were not satisfied, Fisher’s exact test was utilized to provide a more accurate significance assessment. This statistical approach ensured appropriate and robust analysis of the data based on its distribution and characteristics. To explore the effect of stimulation test type on LH peak response, a multivariable linear regression analysis was performed. The model included test type (GnRH vs. triptorelin), age at diagnosis, BMI SDS, IGF-1 levels, and an interaction term between test type and age. Standardized beta coefficients (β) and *p*-values were reported. A two-sided *p*-value < 0.05 was considered statistically significant for all analyses.

This study did not require formal approval from an ethics committee, in accordance with the General Authorization to Process Personal Data for Scientific Research Purposes (Authorization No. 9/2014, Italian Data Protection Authority). According to this regulation, retrospective studies based on anonymized or coded data, in which individual subjects cannot be directly identified, are exempt from the obligation of ethical committee evaluation.

Nevertheless, all procedures and analyses involving patient data were conducted in full compliance with the principles of Good Clinical Practice (GCP) and adhered strictly to the standard diagnostic and management protocols routinely applied in cases of suspected central precocious puberty in our country.

The study was carried out in accordance with the ethical principles outlined in the Declaration of Helsinki and its subsequent revisions. No personally identifiable information was collected, stored, or processed at any stage of the study. Although the nature of the research was retrospective and anonymized, all parents or legal guardians were informed about the study objectives, and written informed consent was obtained from each of them for the use of their child’s clinical data for research purposes.

## 3. Results

**Figure 1:**
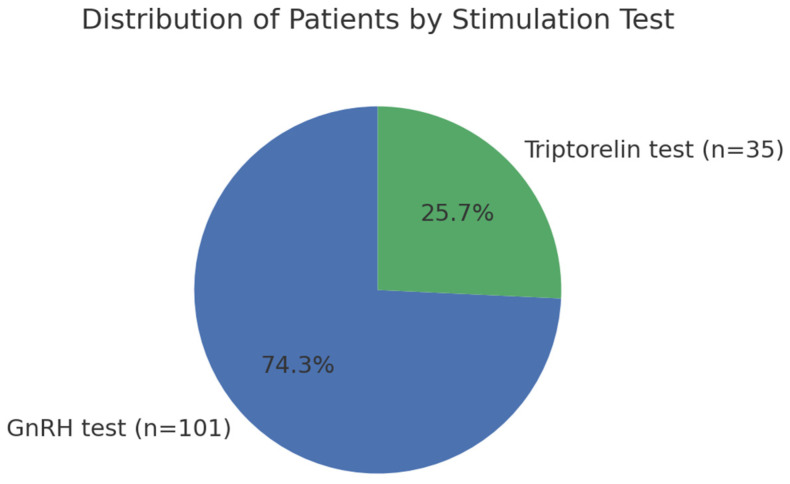
Distribution of female patients with central precocious puberty according to the stimulation test performed. The majority (74.3%) underwent the classical GnRH stimulation test, while 25.7% were evaluated using the triptorelin test.

A total of 136 female patients diagnosed with central precocious puberty were included in the study. Of these, 101 underwent a classical GnRH stimulation test, while 35 were assessed using a triptorelin (triptorelin) stimulation protocol (Figure 1).

The numerical discrepancy between the two groups is explained by the fact that, until recently, the GnRH test was the only test used for the diagnosis of precocious puberty. The use of the triptorelin test has been introduced more recently; therefore, it was not possible to establish a study group of comparable size to that of the GnRH test group, which is larger but also more dated.

Following diagnosis, all patients were treated in accordance with national guidelines using triptorelin therapy, administered either monthly (every 21 or 28 days, depending on clinical characteristics and the physician’s discretion) or every three months.

Anthropometric characteristics such as age at diagnosis, height, height SDS, weight, BMI, BMI SDS, and growth velocity were comparable between the two groups, with no statistically significant differences observed (*p* > 0.05 for all parameters). Similarly, IGF-1 levels did not differ significantly between groups (GnRH: 210 ± 60 ng/mL vs. triptorelin: 208 ± 58 ng/mL; *p* = 0.264).

Regarding gonadotropin levels, all patients (100%) showed a basal LH > 0.1 IU/L; basal LH levels are closely correlated with the LH peak during dynamic stimulation tests, but alone they are not sufficient to confirm precocious puberty. An early activation of gonadotropins, combined with the progression of secondary sexual characteristics, can be an early indicator of CPP and signals the need for further investigations, such as the GnRH test. Using a cutoff of 0.3 IU/L (a threshold value employed in some protocols), we found that 130 out of 136 girls (corresponding to 95.6%) had a value considered pathological.

As expected, significant differences were observed in the gonadotropin response between the two stimulation protocols. Patients who underwent the GnRH test showed a significantly higher peak LH response, particularly at 60 min (9.8 ± 3.1 IU/L), compared to the peak LH at 4 h in the triptorelin group (6.8 ± 2.4 IU/L). Although a direct statistical comparison was not performed due to the different sampling times, the LH response following triptorelin appeared delayed and of lower magnitude.

Moreover, FSH levels were significantly lower in the triptorelin group compared to the GnRH group (3.6 ± 1.3 IU/L vs. 4.2 ± 1.5 IU/L; *p* = 0.004). The LH/FSH ratio was also lower following triptorelin administration (1.9 ± 0.7 vs. 2.3 ± 0.8), although this difference did not reach statistical significance (*p* = 0.129).

Basal LH levels were comparable between groups (1.1 ± 0.6 IU/L in the triptorelin group vs. 1.2 ± 0.7 IU/L in the GnRH group; *p* = 0.933), confirming similar activation of the hypothalamic-pituitary-gonadal axis at baseline. More information is reported in Table 1.

To explore whether the type of test had a differential impact on LH peak depending on the patient’s age, we performed a multivariate linear regression analysis using LH peak as the dependent variable. Independent variables included type of stimulation test, age at diagnosis, BMI SDS, IGF-1 levels, and the interaction term between test type and age.

The regression model revealed that the triptorelin test was associated with a significantly lower LH peak compared to the GnRH test (β = −2.2, *p* = 0.008), after adjusting for age, BMI SDS, and IGF-1 levels. Age at diagnosis was inversely associated with LH peak (β = −0.5, *p* = 0.031), indicating a modest decline in gonadotropin response with increasing age. Notably, the interaction between test type and age was statistically significant (β = 0.6, *p* = 0.022), suggesting that the difference in LH response between the two tests diminishes with increasing age. In other words, the GnRH test elicited a greater LH peak in younger patients, whereas this difference became less pronounced in older girls. BMI SDS and IGF-1 were not significant predictors of LH peak in this model (*p* > 0.1 for both). More information is reported in Table 2.

These findings support the hypothesis that the GnRH test may be more effective in eliciting a robust gonadotropin response in younger patients, and that the diagnostic performance of triptorelin may be more suitable for slightly older children with CPP.

## 4. Discussion

Although basal LH measurement can provide useful preliminary information, it is not sufficient on its own to establish a definitive diagnosis of central precocious puberty [1,2,3]. Basal LH levels may vary according to the timing of sampling, assay sensitivity, and the stage of pubertal activation, leading to possible false-negative results. Therefore, dynamic testing with GnRH or its analogs remains essential for confirming hypothalamic–pituitary–gonadal axis activation, particularly in borderline or early cases [4].

Our retrospective analysis demonstrated that the LH peak achieved during the GnRH stimulation test was generally higher than that observed with the triptorelin test. Furthermore, multivariate statistical analysis revealed that the difference in LH response was more pronounced in younger patients and tended to diminish with increasing age.

The interpretation of the LH peak is a key element in the diagnostic work-up of central precocious puberty, especially in cases with borderline hormonal responses. In fact, for example, an LH peak between 3.3 and 5 IU/L may be interpreted as pathological according to some guidelines, while considered within normal limits by others [7,8,9]. Moreover, although not necessarily indicative of rapidly progressive CPP [7], recent studies have suggested that marked LH responses may be associated with more complex underlying organic conditions [14], such as neoplastic causes of precocious puberty, which are often more challenging to manage clinically [15,16].

To date, the diagnosis of CPP remains multidisciplinary [1], with stimulation tests used to confirm the clinical suspicion [4,5,6,7,8,9]. Recently, some authors have highlighted that the subcutaneous triptorelin test may require a relatively high diagnostic cutoff. In a retrospective analysis of a cohort of 186 girls with premature thelarche, they showed that a lower cutoff, approximately two-thirds of the currently accepted value, maintained excellent sensitivity and specificity for the diagnosis of CPP [17].

A prospective, randomized clinical trial involving 46 girls with premature breast development evaluated the diagnostic accuracy of the subcutaneous Triptorelin test compared to the classical intravenous GnRH test [18]. The results showed that a maximal LH response at 3 h after Triptorelin administration ≥ 7 IU/L (by IFMA) or ≥8 IU/L (by ECLIA) diagnosed CPP with a specificity of 100% (95% CI: 75–100%) and a sensitivity of 76% (95% CI: 58–89%). When combined with estradiol measurements at 24 h, sensitivity increased to 94% (95% CI: 80–99%) and diagnostic accuracy reached 96%. These findings indicate that the Triptorelin test is a highly accurate and valid alternative to the GnRH test, allowing comprehensive evaluation of the pituitary-ovarian axis for differential diagnosis between CPP and precocious thelarche. The triptorelin stimulation test currently appears to be less standardized than the LHRH test, as no universally accepted protocol has yet been established. Although our institutional protocol includes an LH measurement 4 h after stimulation, this sampling point could potentially be omitted in favor of earlier hormonal assessments. A recent study demonstrated that during the triptorelin test, the LH response typically occurs within the first 60 min, and an LH value ≥ 4.52 IU/L at 120 min already provides the highest diagnostic accuracy for CPP [11].

Another recent study reported that the GnRH test and the triptorelin test have comparable efficacy in the diagnosis of central precocious puberty [19]. However, it highlighted that patients undergoing the triptorelin test exhibited higher LH peaks compared to those receiving the GnRH test. This finding contrasts with our results; nevertheless, it is possible that stratifying patients by age could yield different outcomes, potentially aligning with our observations.

Although dynamic tests remain the gold standard for the diagnosis of CPP [1], it is important to comprehensively evaluate the auxological and hormonal characteristics of patients with suspected pubertal activation. Despite their diagnostic sensitivity and specificity, neither the GnRH test nor the triptorelin test can currently be considered fully reliable on their own [20]. The main challenge lies in the absence of a single, universally accepted cutoff value for the GnRH test [7], as well as the lack of clear and standardized protocols for both the administration and interpretation of the triptorelin test [18,19,20,21,22,23].

Our study has some limitations. Its retrospective design may have introduced selection bias and limited the ability to establish causal relationships; the unequal sample sizes between the GnRH and triptorelin groups reflect differences in clinical practice over time but may reduce the statistical power to detect small differences, particularly for variables such as the LH/FSH ratio. Additionally, the timing of LH peak measurement differed between the two stimulation protocols, which precludes direct comparison of absolute LH values and may introduce bias related to pharmacokinetic differences. The diagnosis of central precocious puberty currently requires a multidisciplinary approach, integrating auxological data with radiological findings and detailed hormonal assessments for accurate interpretation.

## 5. Conclusions

Although both the GnRH and triptorelin tests are recognized as valid diagnostic tools for central precocious puberty, our retrospective analysis revealed important distinctions between them. Specifically, the triptorelin test, while effective, requires a longer execution time and is associated with lower LH peak cutoff values compared to the GnRH test. This difference in hormonal response may impact the interpretation of results and subsequent clinical decisions. Pharmacological stimulation tests remain the gold standard for diagnosing CPP; however, the variability in the pharmacological agents employed, as well as the differing hormonal responses elicited by these tests, highlight the complexity of the diagnostic process. Consequently, the diagnosis of CPP cannot rely solely on stimulation test results. Instead, it necessitates a multidisciplinary approach, integrating a thorough evaluation of the patient’s clinical presentation, including auxological data, radiological findings, and comprehensive hormonal assessments.

## Figures and Tables

**Table 1:** Clinical and Hormonal Parameters in Patients Undergoing GnRH or triptorelin Stimulation Test.

Variable	GnRH Test (n = 101)	Triptorelin Test (n = 35)	*p*-Value
Age at Diagnosis (years)	7.35 ± 0.52	7.30 ± 0.50	0.901
Height (cm)	125.50 ± 4.60	125.20 ± 4.80	0.590
Height SDS	1.32 ± 0.55	1.30 ± 0.57	0.660
Weight (kg)	28.90 ± 4.40	28.70 ± 4.20	0.955
BMI (kg/m^2^)	18.30 ± 2.10	18.20 ± 2.00	0.734
BMI SDS	1.05 ± 0.60	1.00 ± 0.62	0.756
IGF-1 (ng/mL)	210.00 ± 60.00	208.00 ± 58.00	0.264
Growth Velocity (cm/year)	7.10 ± 1.20	7.00 ± 1.10	0.327
Basal LH (IU/L)	1.20 ± 0.70	1.10 ± 0.60	0.933
LH peak at 30 min (IU/L)	9.20 ± 3.20	–	–
LH peak at 60 min (IU/L)	9.80 ± 3.10	–	–
LH peak at 4 h (IU/L)	–	6.80 ± 2.40	–
FSH (IU/L)	4.20 ± 1.50	3.60 ± 1.30	**0.004**
LH/FSH ratio	2.30 ± 0.80	1.90 ± 0.70	0.129

**Table 2:** Multivariate Linear Regression for LH peak.

Variabile	Coefficient β	*p*-Value
Test Type (1 = triptorelin)	−2.20	0.008
Test Type × Age Interaction	+0.60	0.022
Age at Diagnosis (years)	−0.50	0.031
BMI SDS	+0.10	0.387
IGF-1 (ng/mL)	+0.02	0.422

## Data Availability

The data that support the findings of this study are available from the corresponding author upon reasonable request.
